# Observational study of clinico-radiological follow-up of COVID-19 pneumonia: a district general hospital experience in the UK

**DOI:** 10.1186/s12879-021-06941-8

**Published:** 2021-12-08

**Authors:** C. A. Musat, M. Hadzhiivanov, V. Durkowski, A. Banerjee, A. Chiphang, M. Diwan, M. S. Mahmood, M. N. Shami, A. Nune

**Affiliations:** Southport and Ormskirk NHS Trust, Southport, PR8 6PN UK

**Keywords:** COVID-19 pneumonia, CXR, British Thoracic Society, Interstitial lung disease, Pulmonary vascular disease

## Abstract

**Background:**

The British Thoracic Society (BTS) recommends that all patients admitted with COVID-19 pneumonia should have a chest X-ray (CXR) and clinical follow-up at 6 or 12 weeks, depending on the disease severity. Little data is available on long-term CXR follow-up for moderate and severe COVID-19 pneumonia. This study aims to evaluate compliance with clinico-radiological follow-up of patients recovering from COVID-19 pneumonia at a local hospital in the UK, as per the BTS guidance, and to analyse radiological changes at clinical follow-up at 12 weeks, in order to risk-stratify and improve patient outcomes.

**Methods:**

This is a single-centre retrospective audit of 255 consecutive COVID-19 positive patients admitted to a local hospital in the UK over 5 months between May and October 2020. All CXRs and clinic follow-up at 12 ± 8 weeks were checked on an electronic database.

**Results:**

Over one in two (131/255) patients had CXR evidence of COVID-19 pneumonia during the initial hospital admission. Half of the patients (60/131) died before CXR or clinic follow-up. Fifty-eight percent (41/71) of the surviving patients had a follow-up CXR, and only two developed respiratory complications- one had residual lung fibrosis, another a pulmonary embolism. Eighty-eight percent (36/41) of the patients had either resolution or improved radiological changes at follow-up. Most patients who had abnormal follow-up CXR were symptomatic (6/8), and many asymptomatic patients at follow-up had a normal CXR (10/12).

**Conclusions:**

Although there were concerns about interstitial lung disease (ILD) incidence in patients with COVID-19 pneumonia, most of our patients with COVID-19 pneumonia had no pulmonary complications at follow-up with CXR. This emphasises that CXR, a cost-effective investigation, can be used to risk-stratify patients for long term pulmonary complications following their COVID-19 pneumonia. However, we acknowledge the limitations of a low CXR and clinic follow-up rate in our cohort.

**Supplementary Information:**

The online version contains supplementary material available at 10.1186/s12879-021-06941-8.

## Background

COVID-19, the first global pandemic since the Spanish flu in 1918, has caused death and devastation worldwide. An outbreak of pneumonia caused by the novel Coronavirus SARS-COV-2 recorded in Wuhan, China, in December 2019 has spread rapidly globally [1]. Viral pneumonia, the main consequence of Coronavirus, has led to a multi-fold increase in hospital admissions and mortality worldwide [2–4]. Pulmonary complications, such as viral pneumonia and pulmonary embolism (PE), are some of the leading causes of death in these patients [5, 6]. Globally, as of the 16th of June 2021, over 177 million confirmed cases of COVID-19, including 3.8 million deaths were reported to the World Health Organisation (WHO) [3].

Peripheral and lower lobe ground-glass lung opacities have been the most common presenting feature of SARS-CoV-2 infection in patients during hospital admission, often evident on chest imaging: CXR (chest X-ray) or CT scan (computed tomography) [1, 6]. There are concerns that patients with COVID-19 pneumonia could potentially develop pulmonary complications such as interstitial lung changes (ILD), pulmonary vascular disease (PVD), pulmonary embolism (PE) and pulmonary arterial hypertension (PAH) [5, 7, 8]. However, long-term data on chest imaging, particularly CXR, is scarce at the moment, with studies focusing more on short-term complications of the disease [9, 10].

The British Thoracic Society (BTS) published their guidance in May 2020 that all patients with COVID-19 pneumonia should be considered for a follow-up CXR at 12 weeks post-viral pneumonia. To evaluate long term respiratory complications, BTS recommended two protocols based on the severity of pneumonia and patients' functional capacity on discharge [11].

The first protocol is for patients with severe pneumonia, admitted to the Intensive Care Unit (ICU), high dependency unit (HDU) or a medical ward. This includes all patients who needed oxygen and those of whom clinicians had concerns at discharge. These patients would benefit from a clinical review remotely at 4–6 weeks and face to face consultation at 12 weeks with a repeat CXR. The second protocol is for patients with mild to moderate clinico-radiological diagnosis of COVID-19 pneumonia who did not require ICU/HDU care. These patients would benefit from a virtual CXR follow up at 12 weeks after discharge to evaluate their recovery.

There is little published data looking at long-term CXR imaging, most data analyses CT chest imaging. As per the BTS guidance, we retrospectively evaluated CXR radiological and clinical follow-up of patients with COVID-19 pneumonia to assess our compliance with this guidance and understand the long-term sequelae following acute disease.

## Methods

This was a retrospective audit of 255 consecutive patients with COVID-19 admitted to a district general hospital in the UK over 5 months, between May and October 2020. All patients were tested positive for SARS-COV-2 on a reverse transcriptase polymerase chain reaction (RT-PCR) assay after a nasopharyngeal swab.

Patients were classified into mild to moderate and severe disease based on the WHO definition of disease severity for COVID-19 [12].

### Mild disease

We included patients with mild COVID-19 symptoms (pyrexia, cough, fatigue, loss of appetite, breathlessness, myalgia, sore throat, headache, diarrhoea, nausea, vomiting, anosmia, ageusia, delirium) without pneumonia and no oxygen requirements. Patients were excluded from the analysis if they had no CXR evidence of pneumonia.

### Moderate disease

Patients with moderate symptoms (fever, cough, shortness of breath), room air SpO_2_ ≥ 90% and chest imaging changes consistent with pneumonia were included.

### Severe disease

We included patients with the signs of pneumonia described above plus either: respiratory rate > 30 breaths/min, room air SpO_2_ < 90%, or with signs of severe respiratory distress (unable to speak in full sentences, accessory muscle use or other general respiratory distress signs).

Electronic clinical notes were checked, and radiology reports for confirmed COVID-19 at the diagnosis and follow-up CXR were included. If there was no available follow-up CXR at 12 ± 2 weeks, earlier or subsequent CXRs were evaluated. We excluded patients who had no CXR at the time of hospital admission or in whom other alternative diagnoses could explain the CXR changes (i.e. heart failure, lung cancer, bacterial pneumonia) or those who died before a follow-up CXR. We checked follow-up appointments for each patient electronically. We used Microsoft Excel to analyse the data.

## Results

**Fig. 1:**
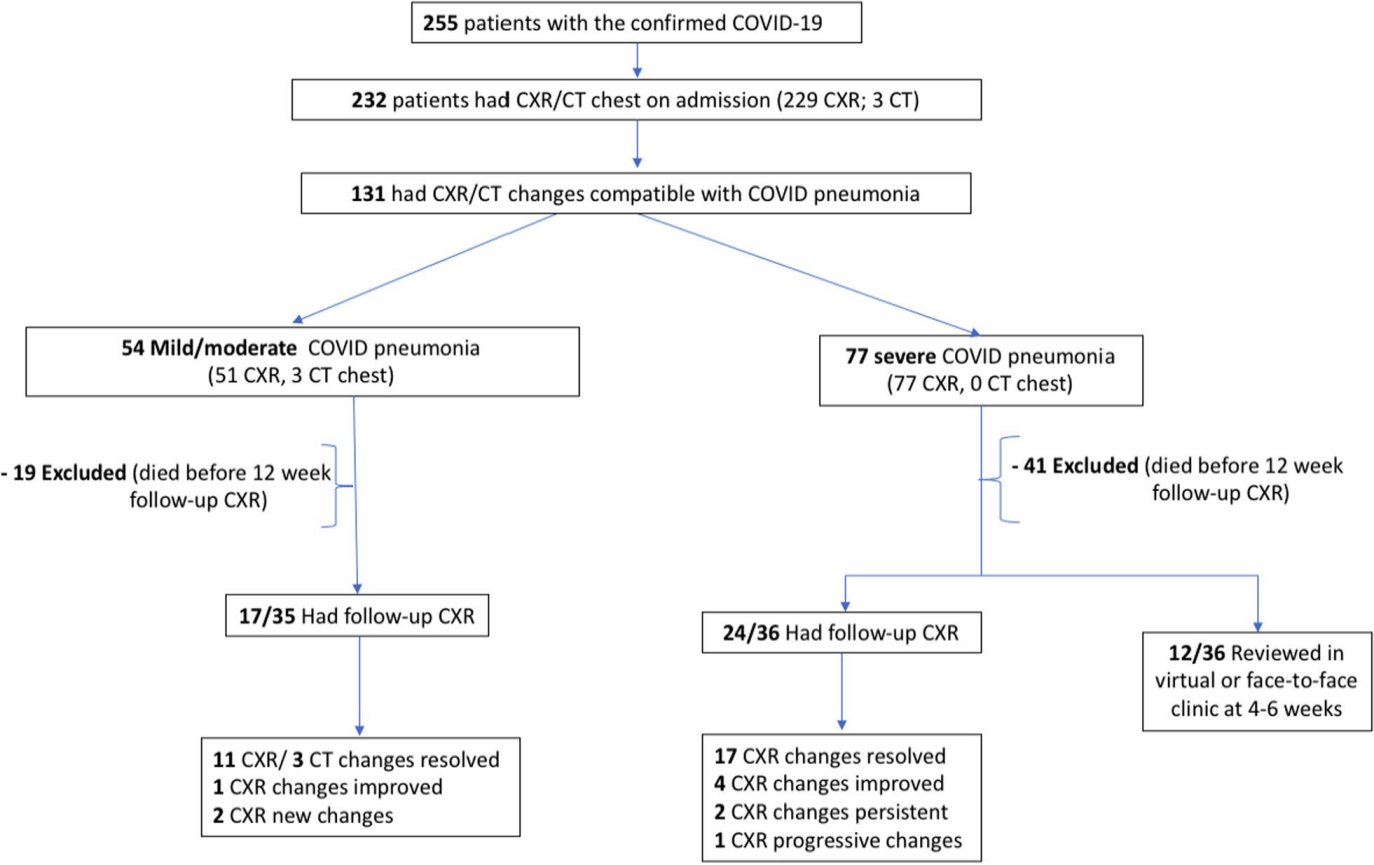
Total number of COVID-19 patients with resolved CXR changes on follow-up

The mean age was 72.9 years, and men slightly outnumbered women (Table 1). The total number of patients admitted to the hospital with COVID-19 between the 1st of May and 10th of October 2020 was 255 (Fig. 1). Of them, 91% (232) of patients had chest imaging (229 CXRs, 3 CTs) at admission. Five patients included in the analysis were readmitted a second time, with worsening COVID symptoms and new CXR changes. Over half (128 CXR, 3 CT) of the patients had radiological changes suggestive of COVID-19 pneumonia during the hospital admission; 54 mild to moderate, and 77 severe viral pneumonia. The overall mortality rate for the COVID-19 positive patients during the hospital admission was 22.7% (58/255). Over 45% (60/131) of the patients who had pneumonia on initial CXR died either during the hospital stay or before the follow-up: 19/54 with mild to moderate pneumonia and 41/77 severe pneumonia. Out of these 60 patients, four were discharged as palliative and died shortly after the discharge.

**Table 1:** Characteristics of the patients admitted to hospital with COVID-19 (n = 255)

Mean age (years) 72.9	Number of cases (%)	
< 60	49	(19.2%)
60–69	37	(14.5%)
70–79	66	(25.9%)
80–89	72	(28.2%)
> 89	31	(12.2%)
Gender		
Male	139	(54.5%)
Female	116	(45.5%)
Ethnicity		
White	234	
Asian	02	
Indian	01	
Black African	01	
Pakistani	01	
Not stated	16	
Hospital stay (in days)		
Mean duration	21.3	
Minimum	01	
Maximum	195	
Outcome-discharged/died		
HDU/ITU admission- Discharged	13	(5.1%)
HDU/ITU admission- Died	08	(3.1%)
Ward level care- Discharged	174	(68.3%)
Ward level care- Died	50	(19.6%)
Ward level care- Palliative on discharge	10	(3.9%)

Forty-one out of 71 (57.7%) surviving patients with COVID-19 pneumonia (17 from mild to moderate pneumonia and 24 from the severe pneumonia group) had follow-up CXR, either arranged at the time of hospital discharge (23) or performed during the same or further hospital admissions (18). At the time of discharge, clinicians arranged follow-up CXR for only 35.9% (28/78) of the patients who had COVID-19 pneumonia and were alive at discharge; three of whom had follow-up booked did not attend, and the other two died before the follow-up. However, another 18 patients had interval CXR during the hospital stay or subsequent hospital admission, which improved the CXR follow-up rate in this study to 58%.

Only 13 patients had a follow-up CXR at the recommended timeline of 12 ± 2 weeks, while 22 patients had it at less than 10 weeks and 6 at more than 14 weeks. All patients who had the CXR at 12 ± 2 weeks had complete resolution of radiological COVID-19 changes (only one had new changes suggestive of bacterial pneumonia). Sixteen of those who had the CXR at less than 10 weeks had resolved radiological changes; therefore, they did not require a further CXR at 12 weeks. Among the 6 patients who had follow-up CXR at more than 14 weeks, three had complete resolution; one had improved lung changes, one persistent lung changes and, another developed new lung changes suggestive of unilateral bacterial pneumonia at 20 weeks (previous COVID-19 pneumonia changes resolved). Table 2 summarises the patients who had remaining or new CXR changes at follow-up.

**Table 2:** Characteristics of patients with abnormal CXRs at follow-up

Case number	Demographics	HDU/ITU admission	Initial CXR Findings	Follow-up CXR findings	Presence of symptoms follow-up	Outcome
1	73, F, WB	No	Left basal consolidation	New right lower zone consolidation (20 weeks), previous changes resolved	Yes, breathlessness	Sent from an OP clinic to hospital, bacterial pneumonia
2	77, M, WB	No	Bilateral lower zone shadowing	Bilateral pulmonary shadowing (17 weeks)	Yes, breathlessness (further hospital admission)	Treated for congestive heart failure
3	41, M, WB	ITU	Bilateral consolidation	Mild persistent bilateral lower lung zone consolidation (8 weeks)	No respiratory symptoms	clear of respiratory symptoms at 6 weeks post follow-up CXR
4	73, M, WB	No	Peripheral, lower zone airspace shadowing	CXR: persistent bilateral airspace shadowing; CT: Progressive severe diffuse interstitial lung changes (4 weeks)	Yes, breathlessness, fatigue, needed palliative oxygen	Rapidly progressive known ILD, started on community palliative oxygen
5	80, M, WB	No	Bilateral mid and lower zone consolidation	Improvement, but residual inflammatory shadowing right lower zone (5 weeks)	Mild breathlessness	No treatment as CXR changes and symptoms improving
6	49, M, Asian	ITU	Bilateral peripheral widespread shadowing	Improvement, minimal shadowing left costophrenic angle and reduction of new fibrotic changes (16 weeks)	Fatigue, insomnia, no respiratory symptoms	discharged from critical care follow-up
7	57, M, Asian	No	Bilateral peripheral shadowing	Improvement, consolidation resolved, persistent mild peripheral bilateral fibrotic changes (6 weeks)	Did not attend clinic follow-up	n/a
8	82, M, WB	No	Left lower zone shadowing	New shadowing left midzone (13 weeks)	Yes (breathlessness, chest pain)	Referred to chest pain clinic, diagnosed as angina
9	76, M, WB	No	Patchy lower zones consolidation peripherally	marked resolution of opacities, remaining atelectatic changes (6 weeks)	no clinical assessment	n/a
10	55, M, WB	ITU	Extensive bilateral opacification (near complete whiteout)	Improvement, only residual bilateral linear infiltrates mid and lower zones (6 weeks)	No respiratory symptoms, reduced mobility	Community physiotherapy, discharged from critical care follow-up

Table summarises demographics, key CXR findings and the presence of symptoms in patients with abnormal CXR at follow-up; *WB* white British, *F* female, *M* male, *OP* out-patient

In total, 31 out of 41 (75%) of patients who had follow-up CXR (14 of mild/moderate and 17 of severe pneumonia) had complete resolution of changes at follow-up, and 5 had significant improvement of changes **(**Fig. 1).

Thirty-three percent (12/36) of the surviving severe pneumonia patients had a clinical review at 5–20 weeks. Half (7/12) of the surviving patients from ICU/HDU stay had no follow-up at 4–6 weeks post-hospital discharge; 3 of these did not attend follow-up. Out of 12 patients who had a clinical review, 6 had resolution of lung imaging changes, 4 had CXR changes at follow-up, 2 had no CXR before clinic (both had mild or absent respiratory symptoms). Five out of 12 patients had resolved respiratory symptoms. Two patients had ongoing mild breathlessness. One patient with mild, new fibrotic changes on CXR had non-specific breathlessness, lethargy, palpitations and muscle aches. After excluding any cardiology cause for her symptoms (normal Transthoracic Echocardiography and 24-h Holter monitoring), the patient was referred to the long COVID clinic and awaiting respiratory input. Another elderly male patient was referred for palliative community oxygen for rapidly progressive pre-existing ILD. Another patient with known bronchiectasis was reviewed by a clinical specialist physiotherapist and given an oscillating positive expiratory pressure device to assist with airway clearance techniques for persistent cough at 10 weeks; one elderly patient had ongoing shortness of breath and reduced exercise tolerance at 8 weeks and was further investigated with CTPA (no PE) and pulmonary function tests (diffusing capacity for carbon monoxide-DLCO 50%). Another patient was reviewed at 2 months post-discharge following PE and had significant improvement in dyspnoea.

In the mild/moderate pneumonia group, 7 individuals had clinic review at 6–20 weeks. Out of these, 3 had complete resolution of lung changes on repeat CXR, 2 had abnormal follow-up CXR, and 2 did not have CXR before the clinic. Three patients had complete resolution of respiratory symptoms at 8, 12 and 16 weeks, respectively. Two patients with known COPD (chronic obstructive pulmonary disease) and asthma had worsening breathlessness at follow-up; one patient with rheumatoid arthritis had exertional breathlessness and fatigue at 6 weeks, which improved at a 10 week follow up. Another patient had breathlessness on exertion at 3 months, diagnosed as angina.

Overall, out of the patients who had abnormal follow-up CXRs (three in the mild-moderate pneumonia group and seven in the severe group), eight had face-to-face or remote consultations and one did not attend their appointment. Six out of these eight patients were symptomatic, with mainly breathlessness and fatigue. On the other hand, from all 12 patients who were asymptomatic at follow-up and who had interval CXR performed, 10 had normal follow-up CXR.

## Discussion

We evaluated the clinico-radiological course of patients with COVID-19 pneumonia following their hospital admission as per the BTS guidance. The main findings of this study were that a total of 76% of patients admitted with COVID-19 pneumonia had complete resolution of CXR changes at follow-up and the majority of asymptomatic patients at follow-up had normal interval CXR. Breathlessness was one of the most common symptoms reported in patients with abnormal CXR at follow-up. In our cohort, a higher proportion of patients with mild/ moderate COVID-19 pneumonia had complete resolution of changes at follow-up (82%), comparing with those with severe pneumonia (71%). Unfortunately, there was a high mortality rate prior to the 12-week review (60/131), which adversely affected the total number of patients for follow-up.

It is reassuring to note that half of the COVID-19 confirmed patients admitted to our hospital had no changes suggestive of COVID-19 pneumonia on initial CXR. Also, contrary to the prediction, most patients with COVID-19 pneumonia in our cohort did not develop pulmonary complications (ILD, PE) during the mean follow-up of 76 days. Only one patient developed mild persistent residual fibrosis on repeat CXR, one had PE 2 weeks after being treated for COVID-19 pneumonia, and two patients had lung collapse during the hospital admission. Ten patients positive for COVID-19 RT-PCR had new mild pleural effusion during admission but not at follow-up.

Like coronaviruses such as Severe Acute Respiratory Distress Syndrome (SARS) and the Middle East Respiratory Syndrome (MERS), SARS-CoV-2 mainly causes respiratory complications. Pulmonary fibrosis, a consequence of lung injury, predominantly due to the severity of the infection and immune-mediated processes, was commonly reported in patients several weeks after acute infection with SARS and MERS [7]. Fibrosis is a manifestation of dysregulation of pulmonary wound repair following acute lung injury and a cascade of inflammatory response leading to irreversible lung architectural deformation. Cytokines from the alternative pathway (IL-4 [interleukin-4], IL-13) and chemokines lead the pro-fibrotic inflammatory response by enhancing fibroblast proliferation, stimulating collagen deposition and inhibiting extracellular matrix degradation [7]. The result is lung scar tissue that appears as a reticular pattern on chest imaging. Likewise, viral and inflammatory components in patients with COVID-19 pneumonia are suspected of causing pulmonary fibrosis. However, there is no sufficient long-term data available to substantiate these assumptions.

SARS and MERS are closely related to the SARS-Cov-2 infection, and therefore, data from previous outbreaks provided a model for COVID-19 pneumonia for monitoring pulmonary complications [2, 11]. In a study of 24 patients with confirmed SARS, 62% of the patients (15/24) had CT evidence of pulmonary fibrosis at 5 weeks after discharge, and 38% (9) had residual ground-glass opacification [13]. In a 12-month follow-up study of 311 SARS positive patients in China, one-fifth (67) had pulmonary fibrosis on HRCT (high-resolution computed tomography) and over a quarter (85) had lung diffusion abnormalities (DLCO < 80% predicted) at the first follow-up at 45.0 ± 20.7 days [14]. Patients with either abnormal pulmonary function or HRCT evidence of pulmonary fibrosis received surveillance HRCT and PFT (pulmonary function tests) every 3 months. It was noted that all (40 in total) patients with abnormal HRCT and PFT had improved fibrosis and lung function at 12 months follow-up [14]. Similarly, in a follow-up study consisting of 36 MERS patients, one third (12) developed lung fibrosis on follow-up chest radiographs arranged at 32–230 days post-discharge. Older age (50.6 ± 12.6 years) and ICU admissions positively correlated with pulmonary fibrosis [15].

Recently published COVID-19 pneumonia patients’ follow-up data is mainly based on CT chest imaging, but very little data is available on follow-up CXRs. A prospective longitudinal study from Wuhan, China, compared interval CT scans for patients discharged from hospital following severe COVID-19 pneumonia (17 ± 11 days and 175 ± 20 days after onset of symptoms) [16]. Over a third (40/114) of the patients had fibrotic-like changes on follow-up CT, 27% (31/114) had residual ground-glass opacities or interstitial lung changes, and over one-third (43/114) showed complete lung changes resolution. In this study, patients with fibrotic-like changes had a higher rate of persistent respiratory symptoms and pulmonary diffusion abnormalities at 6 months follow-up. Similarly, in our cohort, most patients with remaining CXR changes at follow-up had ongoing respiratory symptoms (6 out of 8). Han et al. also showed in their study that older age, tachycardia on admission, longer duration of hospital stay (≥ 17 days), non-invasive mechanical ventilation, and ARDS (acute respiratory distress syndrome) were identified as independent prognostic factors for lung fibrosis at 6-month follow-up [16]. In our cohort, although the mean age of our patients was 73, ILD was not prevalent.

Similarly, a retrospective cohort study of 81 COVID-19 pneumonia patients from India found that almost half (43.2%) of the patients had residual lung changes at 3 months or more (range 90-111 days) on follow-up CT, ground-glass opacity being the most common finding. All patients in this study had CT scan at the time of the hospital admission and at 3 months or over (for those who had persistent symptoms or CXR changes at follow-up), which indicate that only patients with a higher degree of disease severity on admission were analysed [17]. When comparing the persistence of symptoms between the patients with resolved and residual lung findings at follow-up, this study did not demonstrate any statistically significant difference between the two groups. In another study, Zhao et al. evaluated follow-up CT chest at 3 months in 55 patients with moderate COVID-19 pneumonia [18]. They found that two-thirds (71%) had long-term residual radiographic changes on CT, with a quarter of patients having residual lung function abnormalities, although most of the patients were free of respiratory symptoms at follow-up.

In a recently published COVID-19 CXR data, clinicians followed up 73 patients with severe COVID-19 pneumonia admitted to HDU/ICU only. Out of 49 patients who had an interval CXR at 12 weeks, 34 (72%) had complete resolution of lung changes [19]. Similarly, in our study, 76% (31/41) of the patients with COVID-19 pneumonia (moderate and severe) had complete resolution of lung changes on chest radiographs at follow-up.

A few other studies assessed temporal radiographic CXR changes in patients with COVID-19 pneumonia for the short term only [9, 10]. Rousan et al. reported that 13 out of 88 patients hospitalised with COVID-19 had abnormal admission CXR findings. Out of these, 69% (9/13) had either complete resolution or improvement of CXR changes over an average period of 2–3 weeks of hospitalisation [9].

Despite chest CT being regarded as a more sensitive diagnostic tool [20], the American College of Radiologists (ACR) advised that portable CXR may be a better alternative to reduce the risk of cross-infection in radiology suites [21]. Moreover, it is evident from the available literature studies that although CT chest scans are more sensitive in picking up residual lung changes, these patients are relatively asymptomatic. This reiterates that CT chest should be reserved for symptomatic patients with progressive pulmonary disease or those with abnormal CXR at follow-up [11, 21]. Therefore, CXR, a cost-effective investigation, should be used to risk-stratify patients with COVID-19 pneumonia during the follow-up, which is suitable not only in the already overwhelmed UK’s National Health Service (NHS), but also in many parts of the world.

This study emphasises that clinicians should familiarise themselves with the BTS guidance to ensure that patients with COVID pneumonia receive follow-up imaging, best arranged at the time of patient's discharge to evaluate long-term pulmonary sequelae.

Currently, due to the vast volume of available raw data and the fast-paced evolution of the pandemic, the management of COVID-19 patients is evolving. Therefore, more evidence-based studies are needed to understand the clinical journey, long-term sequelae and management of patients affected by COVID-19, to improve their outcomes [22].

### Limitations

The study's main limitation was that relatively a small number of patients had a follow-up CXR.

Lack of Asian and Afro-Caribbean representation in this study could have contributed to the low prevalence of pulmonary complications. Furthermore, the interval between admission and follow-up CXR varied, which could have led to inaccuracies in the total number of abnormal CXR at 12-weeks. Lack of frequent use of echocardiogram or chest CT, particularly for symptomatic patients is also a limitation.

## Conclusion

CXR is a cost-effective and readily available essential investigation tool to evaluate interstitial lung changes following COVID-19 pneumonia. As observed in this study, pulmonary complications seem to be less prevalent in patients with COVID-19 pneumonia than predicted. However, we recommend further extensive studies with a sizeable representation of ethnic minorities to derive firm conclusions about the long-term sequelae of respiratory complications. This will help model guidance for radiology follow-up in patients recovering from COVID-19 pneumonia, with more focus on patients who remain symptomatic, who may need further chest imaging. CT chest remains a favourable diagnostic tool in COVID-19 patients with residual CXR changes and/or physiological impairment. Raising awareness about BTS guidelines for proactive follow-up among primary and secondary care clinicians is essential to increase the pick-up rate of patients with ongoing radiological changes on follow-up chest imaging for COVID-19 pneumonia.

## Supplementary Information

**Additional file 1:** Raw data used for study analysis with demographics, chest X-ray and clinic follow-up details.

## Data Availability

All data generated or analysed during this study are included in this published article [and its Additional file 1]. All methods were carried out in accordance with relevant guidance and regulations.

## Abbreviations

CXR: Chest X-ray
CT: Computed tomography
ILD: Interstitial lung disease
PVD: Pulmonary vascular disease
PE: Pulmonary embolism
PAH: Pulmonary arterial hypertension
ICU: Intensive Care Unit
HDU: High dependency unit
RT-PCR: Reverse transcriptase polymerase chain reaction
WHO: World Health Organisation
WB: White British
F: Female
M: Male
OP: Outpatient
CTPA: CT pulmonary angiogram
DLCO: Diffusing capacity for carbon monoxide
COPD: Chronic obstructive pulmonary disease
SARS: Severe Acute Respiratory Distress Syndrome
MERS: Middle East Respiratory Syndrome
IL: Interleukin
HRCT: High-resolution computed tomography
PFT: Pulmonary function tests
ARDS: Acute respiratory distress syndrome
ACR: American College of Radiologists
NHS: National Health Service
